# Evolution of Corrosion Products Formed during the Corrosion of MgZr Alloy in Poral Solutions Extracted from Na-Geopolymers Used as Conditioning Matrix for Nuclear Waste

**DOI:** 10.3390/ma13214958

**Published:** 2020-11-04

**Authors:** Rémi Boubon, Xavier Deschanels, Martiane Cabié, Diane Rébiscoul

**Affiliations:** 1 ICSM, CEA, CNRS, ENSCM, Université de Montpellier, Marcoule, 30207 Bagnols-sur-Cèze, France; remi.boubon@cea.fr (R.B.); xavier.deschanels@cea.fr (X.D.); 2 CP2M, Aix-Marseille Université, 13397 Marseille, France; martiane.cabie@univ-amu.fr

**Keywords:** magnesium alloys, X-ray Diffraction, TEM, alkaline corrosion, passive film evolution, interfaces, geopolymer

## Abstract

Geopolymer, a nanoporous aluminosilicate filled with water and ions, has been selected as a potential matrix to encapsulate MgZr alloy fuel cladding. In this study, we investigate the evolution of the corrosion products formed during the corrosion of MgZr in poral solutions extracted from geopolymers with and without NaF as corrosion inhibitor. Using various characterization techniques such as Scanning Electron and Scanning Transmission Electron Microscopies coupled to Energy Dispersive X-ray spectroscopy and Grazing Incidence X-ray Diffraction, we show that the amounts of dissolved silica and fluoride species in solution are the key parameters driving the nature of corrosion products and probably their passivating properties regarding MgZr corrosion.

## 1. Introduction

The dismantling of the Uranium Natural Graphite Gas reactors (UNGG) in France had generated a large amount of magnesium waste coming from the fuel cladding. These fuel claddings made of MgZr 0.5 wt.% alloy [1] were classified as intermediate-level radioactive waste due to the uranium and fission products residues remaining after the spent nuclear fuel removal. Mineral binder such as geopolymer, a nanoporous aluminosilicate filled with water and ions [2], was selected as a potential matrix to encapsulate these wastes. This matrix presents three main advantages regarding the corrosion of MgZr that can limit the H_2_ formation that is detrimental to the safety of the nuclear repository. First, for pH > 10.5, the corrosion of Mg and magnesium alloys leads to the formation of a stable passive film of brucite Mg(OH)_2_ [3,4]. As the pH of the poral solution coming from geopolymers is around 12, this passive film may be formed, which would explain the decrease of the corrosion rate of Magnesium alloys embedded [5,6]. Second, the presence of dissolved silica within the poral solution is also an asset since it was demonstrated that formation of magnesium silicates by an anodization process on the surface of magnesium improve its resistance regarding its corrosion [7,8,9]. In addition, some authors have shown that silicate sol–gel coating on Mg substrate induces reduction on corrosion [10,11]. Third, it is possible to integrate a corrosion inhibitor such as NaF in poral solution [6]. The presence of fluorine species leads to a decrease of magnesium corrosion [12] by the possible formation of several phases such as MgF_2_, KMgF_3_, NaMgF_3_ and maybe Mg(OH)_2−*x*_F*_x_* [13,14,15,16]. Some authors have shown that Mg-silicates or phases incorporating fluorine are more passivating than Mg(OH)_2_ [17,18]. Generally, MgZr corrosion tests were carried out in activation solutions from geopolymers, in synthetic solutions or embedded in geopolymers. These tests are performed using electrochemical experiments such as galvanic corrosion using steel [19] and corrosion potential measurements [5,18], both coupled with H_2_ release measurement in order to determine the corrosion rate of MgZr. Some results showed that fluoride in solution reduced the corrosion rate of MgZr by a factor 2, compared to the NaOH solution with silicate thanks to a synergic effect between silicate and fluoride [19]. Indeed, the presence of such species in solution may lead to the formation of magnesium silicates and fluorine compounds that passivate the MgZr surface as detected by EDX analysis [5]. Recently, using electrochemical experiments of MgZr corrosion within geopolymer, it has been highlighted that the presence of NaF leads to the formation of corrosion products like MgF_2_ and NaMgF_3_ having various passivating properties. They also demonstrated that fluoride compounds had different passive properties depending on NaF concentration in solution [18]. However, in this study, no magnesium silicate was detected, which is not the case in [19].

In this work, we propose to highlight the nature and the morphology of the various corrosion products formed at the surface of MgZr alloy during its natural corrosion in poral solutions with and without the corrosion inhibitor. To reach this goal, we studied the evolution over one year of the corrosion products (CP) present at the surface of MgZr during its corrosion in poral solutions (PS) extracted from geopolymers with and without NaF as corrosion inhibitor at ambient temperature. Using various characterization techniques such as Scanning Electron and Scanning Transmission Electron Microscopies coupled to Energy Dispersive X-ray spectroscopy (SEM-EDX and STEM-EDX) and Grazing Incidence X-ray Diffraction (GI-XRD), we determined the nature and morphologies of CP. For the first time, we also demonstrated that the amounts of dissolved silica and fluoride species in solution are the key parameters driving the nature of CP.

## 2. Materials and Methods

### 2.1. Materials and Solutions

Magnesium alloys (Magnesium: 99.5% and Zirconium 0.5 wt.%) ingots of 100 × 120 × 10 mm^3^ used in this study were supplied by Neyco Society (Vanves, France) [5]. The impurities are given in the Table 1. No secondary phase was detected in the microstructure of this alloy. In the following study, this alloy will be referred as MgZr.

MgZr substrates of 20 × 10 × 3 and 5 × 5 × 3 mm^3^ were cut from ingots using a diamond wire saw of 150 µm at 0.8 rpm under ethanol as lubricant. Afterwards, samples were polished with SiC papers having various grades (500 and 1200) and diamond suspensions of 9 and 3 µm on clothes (MD-Largo and MD-Dac) using ethanol as lubricant. Final polishing was performed using vibrational polishing with a colloidal silica solution of 40 nm mixed with 50 vol.% of ethanol on MD-NAP (Struers). After polishing, samples were cleaned by ultrasound in ethanol for 15 min, rinsed using ethanol, dried under N_2_ and saved in an Ar glove box to avoid potential oxidation.

For the poral solutions extraction, two geopolymers named GP and NaF-GP were synthetized in two steps as reported in [2,6]. The target molar ratio of the GP and NaF-GP geopolymers was 1Na_2_O-3.96SiO_2_-1AlO_2_-12.5H_2_O. First, an activation solution was prepared by dissolving 16.92 g of sodium hydroxide (NaOH pellets, Sigma-Aldrich, Saint Quentin Fallavier, France, purity 99.9%) in 3.68 mL of ultrapure water and 130.04 g of a commercial sodium silicate solution (Betol 39T, Woellner, Nogent L’Artaud, France, 27.80 wt.% of SiO_2_, 8.30 wt.% of Na_2_O and 63.90 wt.% of H_2_O) under magnetic stirring for one hour. The dissolution of silicate being exothermic, the activation solution was cooled down to room temperature. Second, 102.44 g of metakaolin (Argical-M-1000 from AGS Mineraux, Clérac, France, 54.40 wt.% of SiO_2_, 38.40 wt.% of Al_2_O_3_ and 7.2 wt.% of impurities) was added to the activation solution and stirred for 10 min until homogenization. In order to obtain a geopolymer containing fluoride ions, sodium fluoride (NaF, 99%, Strem Materials, Bischheim, France) solution at 1.25 M (0.012 wt.%) was added to the activation solution and the solution was stirred during 1 h before adding the metakaolin.

Afterwards, 5 to 10 mL of poral solutions referred as PS and NaF-PS solutions were extracted from these geopolymers using a high-pressure device as in [20] reaching an axial pressure of 300 MPa at 3 min at 2.4 kN·s^−1^. Poral solutions were filtered using a membrane filter having a 0.25 µm pore size, acidified to 2 vol.% of HNO_3_ (65 wt.%, Suprapur Merck, Fontenay sous Bois, France) and then analyzed using ICP-AES for Si and Na ions. Fluoride and chloride specific electrode (perfectION^TM^ from Mettler-Toledo, Viroflay, France) were used to determine F and Cl concentrations. The pH and the elementary composition are presented in Table 2.

### 2.2. Corrosion Experiments

**Figure 1:**
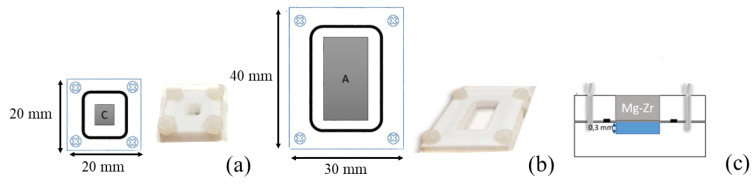
Description of the reactors used for the MgZr substrates corrosion in poral solutions extracted from geopolymers. (**a**) Reactor for samples analyzed using microscopies (5 × 5 × 3 mm^3^), (**b**) reactor for samples analyzed by GI-XRD (20 × 10 × 3 mm^3^) and (**c**) cross section schematization of the reactor.

MgZr substrates corrosion tests were performed in PTFE reactors, presented in Figure 1, at 20 °C with a MgZr surface area-to-poral solution volume ratio S/V of 30,000 m^−1^ at 20 °C. This S/V was chosen to have the highest S/V (S/V existing at the interface between MgZr and a pore of the geopolymer is about 10^10^ m^−1^, see calculation in SI) allowing the limitation of the evaporation of the solution in contact with MgZr samples. After the reservoir were filled with poral solutions (few droplets needed), MgZr substrates of 20 × 10 × 3 and 5 × 5 × 3 mm^3^ dedicated to GI-XRD and SEM-EDX, respectively, were embedded in the PTFE holder using a viton seal and screwed on top of reservoirs containing the solution (Figure 1). Then, experimental set-ups were stored in a glove box under an argon atmosphere in plastic bags at 90% of relative humidity to avoid solution evaporation. After each MgZr substrate sampling for characterization at 1, 7, 14, 30, 90, 180 and 360 days, the small amount of leachate was completely replaced by fresh poral solution. Each sample was rinsed with ethanol, ultrapure water and then again with ethanol and prepared in a glove box before their analyses. Afterwards, the samples were put back in contact with fresh poral solution.

### 2.3. Solid Characterizations

GI-XRD measurements were performed using a Bruker (Champs-sur-Marne, France) D8 diffractometer with primary optics in Bragg–Brentano geometry. This apparatus is equipped with a copper tube (Cu Kα_1,2_λ = 1.54 Å) operating at 40 kV and 40 mA. Data were collected between 5° and 50° (2θ mode) with a 0.02° step. Such measurements were performed at incidence angle θ*_i_* of 0.2° and 2° in order to probe the samples at two different depths. Indeed, the penetration depth of the X-ray beam P(θi) (nm) depends on the absorption coefficient β (cm^2^·g^−1^) and the mass density ρ (cm^3^·g^−3^) of the materials. For θ*_i_* = 0.2°, P(0.2) is around few hundred nanometers and for 2° P(2) is of few micrometers (see calculation in SI 1, Figure S3). Before analysis, the sample was placed in an experimental set-up in a glove box allowing the analysis under N_2_ atmosphere (Figure S4).

Samples were analyzed by Scanning Electron Microscopy (SEM) with an FEI (Hillsboro, OR, USA) Quanta 200 environmental scanning electron microscope using a back-scattered electron detector or a secondary electron detector in vacuum conditions with an acceleration voltage of 5 kV. Energy-dispersive X-ray spectroscopy (EDX) semi-quantitative cartographies were performed during 20 min for each analysis.

For the thin foil lamella, a protective platinum layer is deposited on the top of the CP layer to avoid degradation during preparation. After the preparation of thin foil (approximately 200 nm), by dual-beam FIB using FEI Helios 600 192 NanoLab, samples were analyzed by TEM and STEM-EDX. STEM and TEM observations were performed on an FEI Tecnaï G2 equipped with a LaB6 source operating at 200 kV. The detectors were a Gatan CCD camera, a STEM BF-DF detector and a SDD Detector (X-Max80, Oxford Instruments, Gometz la ville, France) for the EDX analyses. Quantification was done by using k-factor method from Cliff-Lorimer with standard and neglecting/ignoring absorption. Analysis was performed on the rectangle for 30 s to avoid degradation of CP layer such as hole formation or dehydratation of CP [21,22].

## 3. Results

### 3.1. Morphology and Composition of the CP Layers

**Figure 2:**
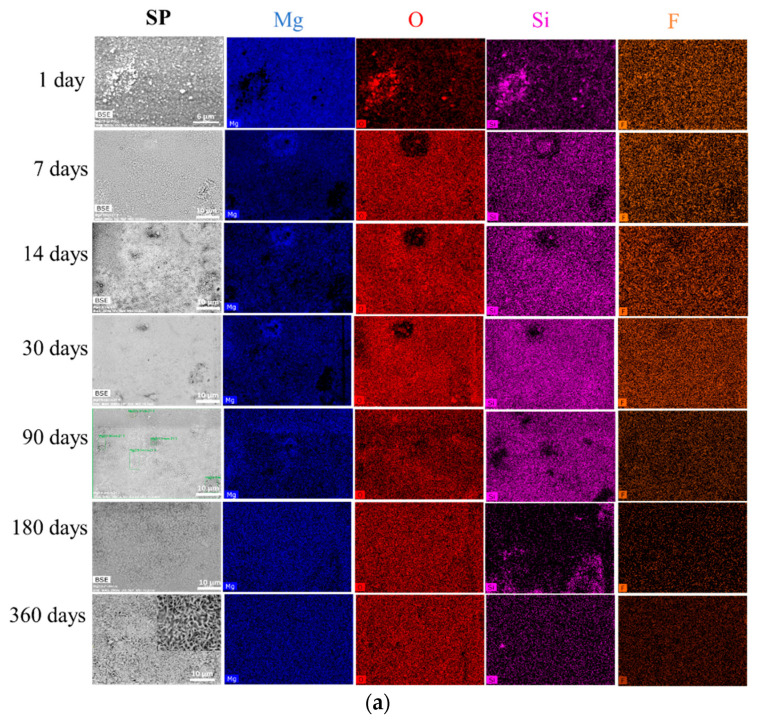

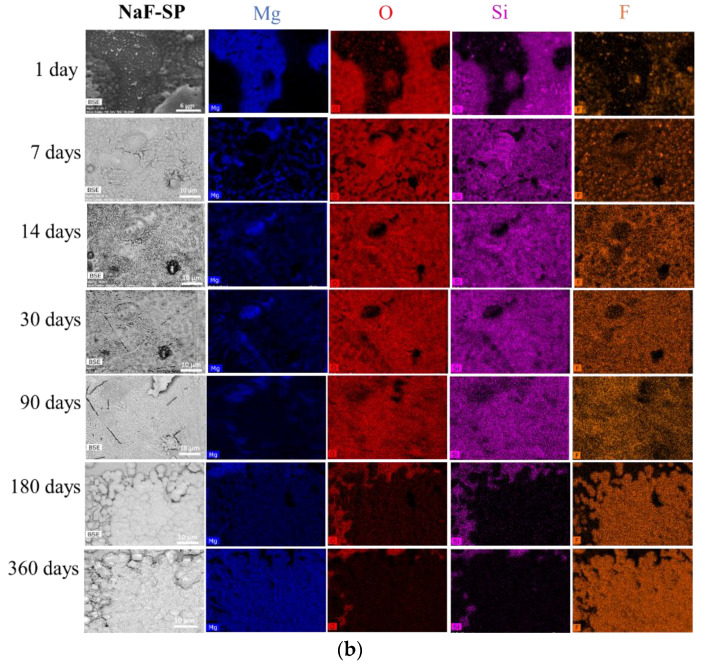
SEM images of the corroded MgZr substrates in (**a**) poral solution (PS) and (**b**) NaF-PS as a function of corrosion time and corresponding EDX cartographies.

The Figure 2a,b present the evolutions of the MgZr substrate surface as a function of corrosion time in PS and NaF-PS and the associated elementary cartographies obtained by SEM-EDX. The presence of salts and carbonates after 1 and 7 days of corrosion is linked to a possible precipitation of these phases during the MgZr substrates sampling (Figure S5a). The later samples (corrosion time > 14 days) were rinsed following the procedure described in the experimental section from 14 days.

**Figure 3:**
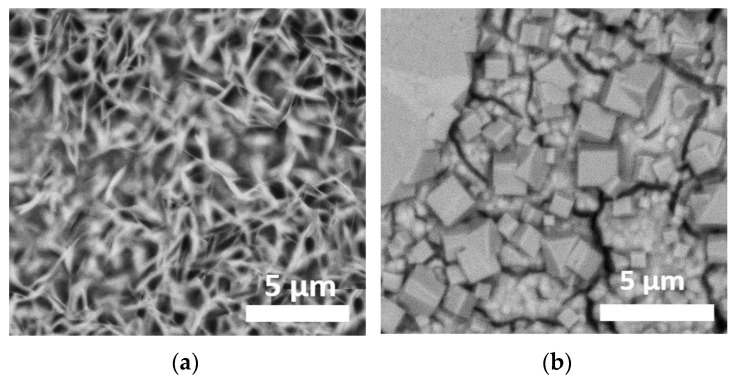
SEM images of the corroded MgZr substrates at 360 days in (**a**) PS and (**b**) NaF-PS.

Figure 3 presents images of higher magnificence of the MgZr surface for (a) PS and (b) NaF-PS at 360 days.

#### 3.1.1. MgZr Corroded in PS

First, we have to notice that the few silica particles coming from the last polishing step present on sample surface are not visible after 1 day of corrosion. Their dissolution and/or the dissolution of the MgZr localized underneath may explain their removal. Second, as illustrated by Figure 2a, the surface of MgZr substrate presents a deposit from 7 to 90 days. As shown by the EDX maps, during the first 90 days, the surface consists mainly of Mg, O, Si and F (maps of Na and C are presented in Figure S5, see also EDX spectra in Figure S6). The presence of F comes from its low concentration in the PS (Table 2) and thus, its possible integration within the CP layer. C probably comes from the formation of a slight amount of carbonates during the transfer of the sample for analysis, or it could be due to a contamination of the surface during the SEM analysis. At 180 and 360 days, the signal of Si is less intense on the surface and only Mg and O are detected (see also EDX spectrum in Figure S6). This phenomenon is associated with the formation of the crystalline phase having a platelet shape that is typical of brucite [23] (see Figure 3a). This phase covers the main part of the sample surface.

**Figure 4:**
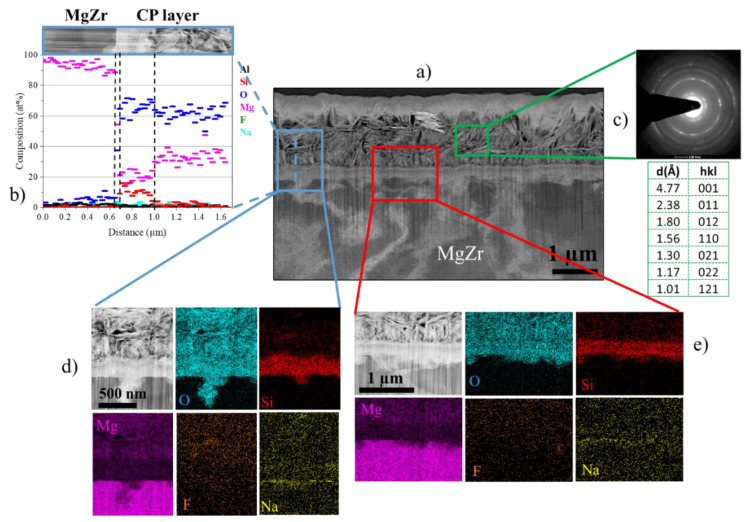
(**a**) Image of corrosion products (CP) layer of MgZr substrate corroded in PS during 360 days in DF-STEM mode, (**b**) elementary profile obtained in CP layer from EDX analysis (blue rectangle), (**c**) electron diffraction of the phase having platelet shape and identified as brucite Mg(OH)_2_ (green rectangle), (**d**,**e**) EDX cartographies of two zones (red and blue rectangles).

The results obtained at the corroded MgZr surface at 360 days are confirmed by the STEM-EDX analysis of the thin foil presented in Figure 4.

Figure 4a highlights a CP layer of 1.46 ± 0.2 µm with the presence of local corrosion. This CP layer presents several zones marked by the dotted line in Figure 4b with various compositions. The first one is a thin zone of a few tens of nanometers located at the MgZr surface of Mg and O with a few amount of Si. The second zone of 400 nm consists of Mg, O and Si corresponding to the composition characterized by SEM-EDX at the surface of the sample up to 90 days. The third one is a zone of 1 µm in contact with PS-12.5 presenting platelet crystallites with Mg and O. The electronic diffraction analysis points out a polycrystalline brucite phase as confirmed by the indexing of the (hkl) distances of this zone 3 (Figure 4c). This observation goes with the SEM-EDX analysis and attests that only brucite is formed from 90 days and grows until the last day of analysis (360 days).

**Figure 5:**
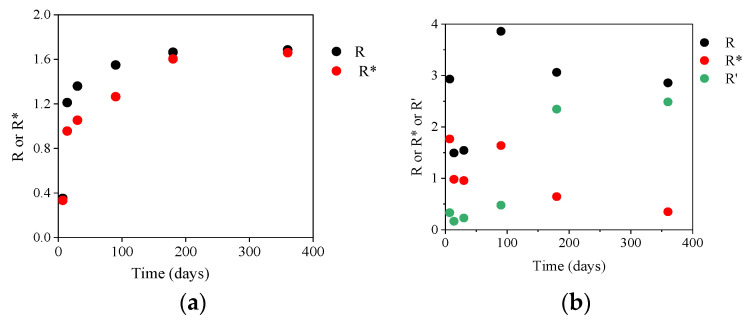
Evolutions of the ratio R = I_O_/I_Mg_ and R* = I_O_/(I_Mg_ + I_Si_) for SP or R = (I_O_ + I_F_)/I_Mg_, R* = (I_O_ + I_F_)/(I_Mg_ + I_Si_) and R’ = I_F_/I_Mg_ for NaF-SP as a function of the corrosion time of MgZr substrate in (**a**) PS and (**b**) NaF-PS.

Likewise, the comparison of the evolution of the ratios of the maximum of peak intensities of O on the maximum of peak intensities of Mg and/or Si, R (R = I_O_/I_Mg_) and R* (R* = I_O_/(I_Mg_ + I_Si_)) obtained from the cartographies in Figure 2 and presented in Figure 5a confirms these results. Indeed, R and R* increase as a function of time and tend toward 2. Moreover, the difference between R and R* decreases until 180 days (with R > R*). A few hundred nanometers depth of CP layer had been probed by surface EDX analysis, this means that magnesium silicates having R* comprised between 1.33 and 1.80 were mostly formed until 90 days and that, afterwards mainly brucite forms.

#### 3.1.2. MgZr Corroded in NaF-PS

The SEM-EDX analyses of the surface of the MgZr substrate corroded in NaF-SP solution presented in Figure 2b highlight the presence of a deposit consisting of O, Mg, F and Si (Na and C maps are presented in Figure S5b) at the surface of MgZr from 14 to 90 days of corrosion. From 180 days, the sample surface presents some spherical particles partially covered by smaller crystallites. At 360 days, these crystallites consisting of Na, Mg and F have increased. They present a cubic shape as it is clearly seen in Figure 3b. The surface, not covered by these particles, has a composition similar to that for the previous time (Figure S7).

**Figure 6:**
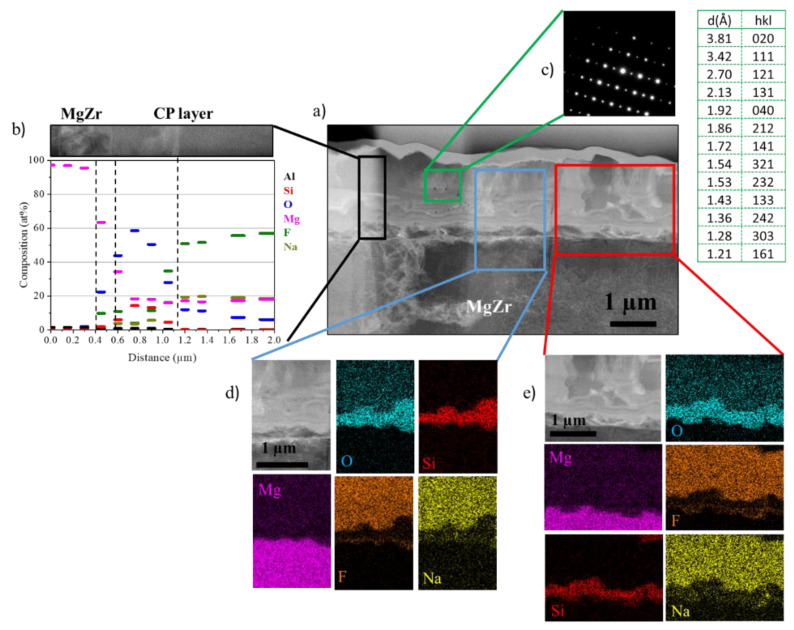
(**a**) Image of CP layer of MgZr substrate corroded in NaF-SP during 360 days in DF-STEM mode, (**b**) elementary profile obtained in CP layer from EDX analysis (black rectangle), (**c**) electron diffraction of the phase having cubic shape identified as NaMgF_3_ (green rectangle), (**d**,**e**) EDX cartographies of two zones (red and blue rectangles).

The results obtained at 360 days are confirmed by the STEM-EDX analysis of the thin foil of the corroded MgZr in NaF-SP presented in Figure 6.

The results obtained shows that CP layer formed in NaF-SP during 360 days presents a buried MgZr/CP layer interface and a thickness around 1.60 ± 0.12 µm (Figure 6a). As for MgZr corroded in PS, this CP layer presents several zones with different compositions. The first zone of 200 nm in contact with MgZr consists of Mg, O and F, which may be Mg(OH)_2−*x*_F*_x_*. Indeed, Mg(OH)_2−*x*_F*_x_* can be formed by the isomorphic substitution of OH^−^ by F^−^. The second zone is about 300 to 500 nm thick and it is composed of Mg, O and Si with a small fraction of F. This phase may be associated to magnesium silicates partially substituted by F. The third zone of 1 µm, corresponding to the cubic phases in surface analysis, mainly consists of Na, Mg, F and O. The electron diffraction analysis shows the presence of NaMgF_3_ crystallites (Norbergite) (Figure 6c).

**Figure 7:**
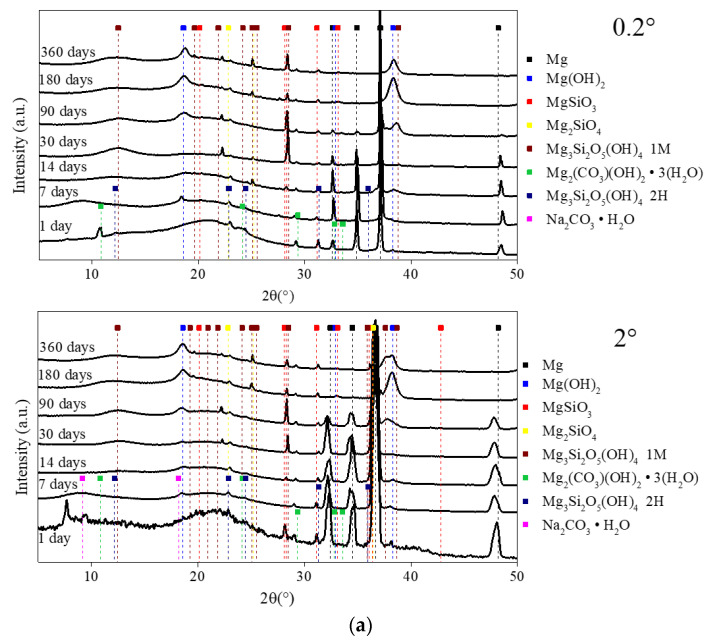

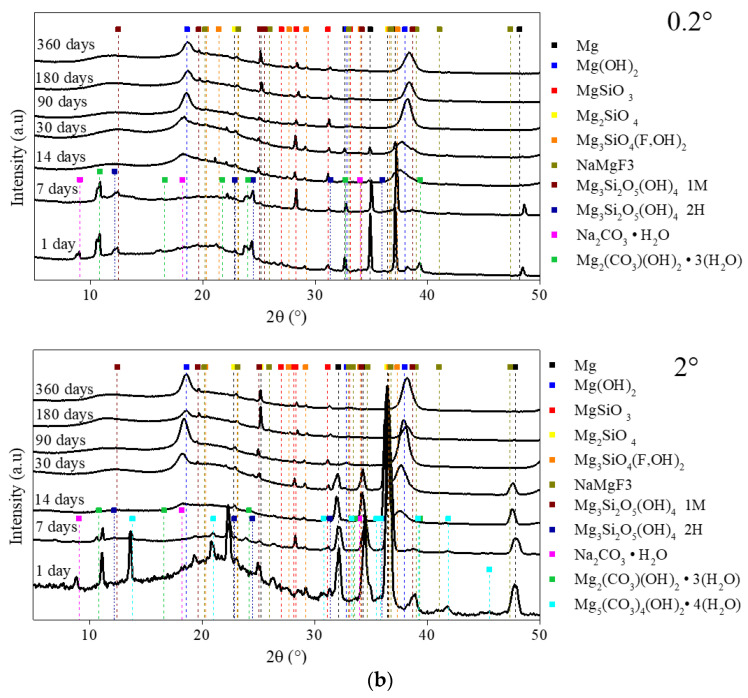
GI-XRD patterns as a function of the corrosion duration of MgZr in (**a**) PS and (**b**) NaF-PS recorded at θ*_i_* = 0.2° and 2°.

The formation of these zones are correlated with the comparison of the evolutions of following ratios R (R = (I_O_ + I_F_)/I_Mg_), R* (R* = (I_O_ + I_F_)/(I_Mg_ + I_Si_)) and R’ (R’ = I_F_/I_Mg_) (obtained from the cartographies in Figure 2b and presented in Figure 5b. The first two zones formed from 14 to 30 days of corrosion have ratios R = 1.5 and R* = 1 which may correspond to the presence of Mg(OH)_2−*x*_F*_x_* and magnesium silicates. At 90 days, R is close to 4, R* = 1.6 and R’ = 0.8. These values may highlight the presence of magnesium silicates with fluoride substitution such as those determined by GI-XRD (Figure 7b): MgSiO_3_, Mg_3_Si_2_O_5_(OH)_4_ and Mg_3_SiO_4_(F,OH)_2_ whose R* are 1.5, 1.8 and 1.25, respectively. From 180 days, R’ increases to reach 2.3 and O/Mg = 0.7 attesting NaMgF_3_ formation and the presence of a small amount of Mg(OH)_2−*x*_F*_x_*.

### 3.2. Determination of the Evolution of Crystalline CP by GI-XRD

#### Nature of CP

Figure 7a,b present the evolution of the X-ray diffraction patterns obtained by GI-XRD of the corroded MgZr substrates of 20 × 10 × 3 mm^3^, which is different from previous analyses, as a function of time in PS and NaF-PS.

These results highlight that whatever the samples and θ*_i_*, the peaks attributed to the Mg hexagonal phase (ICDD-JCPDS card No. 01-082-9643) decrease the attesting of CP formation on the surface of MgZr. Between 0.2° and 2°, the only noticeable difference is the peak’s disappearance of Mg from 90 days for θ*_i_* = 0.2° and from 180 days for θ*_i_* = 2°. This can be explained by the thickness of the CP layer being higher than the depth probed by the beam. Moreover, the XRD patterns of the corroded MgZr highlight the formation of several crystalline CP. The presence of salts and carbonates such as Na_2_CO_3_·H_2_O (Rruff No R050499), Mg_2_(CO_3_)(OH)_2_·3(H_2_O) [24] and Mg_5_(CO_3_)_4_(OH)_2_·4H_2_O [24] at 1 and 7 days of corrosion is linked to the probable precipitation of these phases during the MgZr substrates sampling as observed by SEM-EDX (Figure 2a,b). A broad pic between 12° and 19° attesting the amorphous or semi-crystalline phase formation is also visible in all analyses.

**Figure 8:**
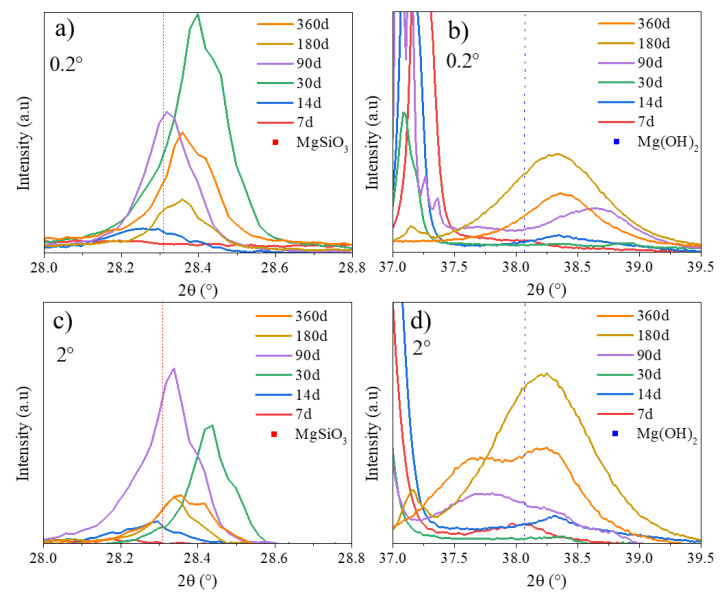
Zoom of XRD patterns of the zone between 28° and 28.8° corresponding to the peak of MgSiO_3_ at (**a**) 0.2°-PS and (**c**) 2°-PS and a second zone between 37° and 39.5° corresponding to the peak of brucite at (**b**) 0.2°-PS and (**d**) 2°-PS.

In PS, brucite (ICDD-JCPDS card No. 01-071-5972) and magnesium silicates such as Mg*_x_*SiO*_y_*(OH)*_z_* (MgSiO_3_: Rruff No. R040093; Mg_2_SiO_4_: Rruff No. R040057; Mg_3_SiO_5_OH_4,_1M: ICDD-JCPDS card No. 00-022-1164 and Mg_3_SiO_5_OH_4,_2M from [25]) are present in all the CP layers since they are visible at 0.2° and 2°. The evolution of the brucite in CP layer may be associated with the formation of the magnesium silicates. Indeed, the peak at 28° of MgSiO_3_ appears at 30 days and then decreases from 90 days. This is associated with the significant increase of the intensity of the peaks attributed to brucite at 2θ = 18.5° and 38.1° from 90 days as displayed in Figure 8a,b. This result confirms the result presented previously indicating the presence of brucite on the surface of MgZr, of magnesium silicates and of crystallized brucite above.

For the MgZr substrate corroded in NaF-PS, the XRD patterns highlight the formation of brucite since 14 days and of magnesium silicates such as in PS but also magnesium silicates fluoride (Mg_3_SiO_4_(F,OH)_2_: ICDD-JCPDS card No. 01-074-0993). A peak of low intensity attributed to NaMgF_3_ (ICDD-JCPDS card No. 01-070-3874) is also visible at 360 days.

### 3.3. Brucite Evolution during MgZr Corrosion

Being the main phase appearing during the corrosion layer formation, the evolution of brucite’s crystallite size has been determined. Because the intensity of the diffracted signal under grazing incidence is too low to allow Rietveld refinement of the patterns, the crystallite size D of brucite formed during the corrosion of MgZr in PS and NaF-PS was estimated using the Scherrer formula (1) using the most intense peak at θ_011_.

(1) $$D=\frac{K\times \lambda }{\beta \times \cos\left(\theta \right)}$$

with *K*, the Scherrer constant fixed at 0.9; λ(Å), the wavelenght of X-ray beam (*K*α1(Cu) = 8 keV = 1.54 Å), β(rad), the width at half maximum; and θ(rad), the peak position. β was determined fitting the peak at θ_011_ with a Gaussian function.

**Figure 9:**
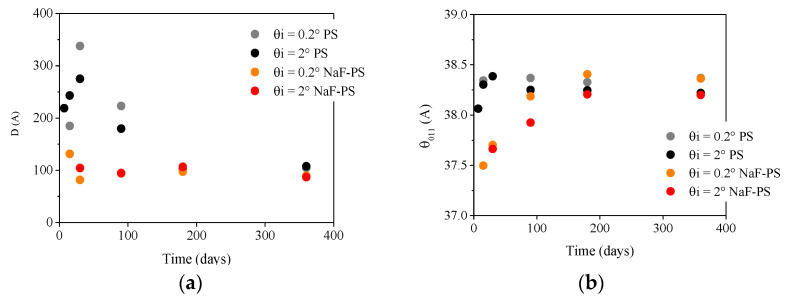
Evolutions of (**a**) D and (**b**) θ_011_ of brucite as the function of corrosion duration of MgZr substrates in PS and NaF-PS.

D was calculated from GI-XRD patterns obtained from 7 days in PS and from 14 days in NaF-PS. The D evolution is presented in Figure 9a. The θ_011_ peak position was also investigated in order to determine a modification of the cell volume. Its evolution with the corrosion duration is displayed in Figure 9b.

The evolutions of D and the position of peak θ_011_ of brucite varies with time as a function of the poral solution. First, the calculated crystallites’ size is larger when brucite is formed in PS than in NaF-PS during the first 90 days. Second, while θ_011_ is almost constant (38° < θ_011_ < 38.4°) as a function of the MgZr corrosion duration in PS, in NaF-PS, θ_011_ increases from 37.5° and tends toward a value between 38.2° and 38.4° depending on the incident angle θ*_i_*. This indicates a modification of the cell volume linked to the isomorphic substitution of OH^−^ by F^−^ leading to the formation of Mg(OH)_2−*x*_F*_x_* [15,26]. Third, for MgZr corroded in PS, the D evolution presents two stages. During the first stage, D increases until 30 days and D is higher for brucite located on the surface (θ*_i_* = 0.2°) than deeper in the CP layer (θ*_i_* = 2°). After this corrosion duration, D decreases to 100 Å. This D decrease is associated with the intensification of the magnesium silicates peaks at 30 days.

Finally, for corroded MgZr in NaF-PS, D slightly decreases from 14 to 30 days and afterwards remains close to 100 Å. This decrease may be also linked to magnesium silicates formation but in a shorter corrosion time. Moreover, for this sample, only a peak of NaMgF_3_ having a low intensity at 360 days is detected. Thus, its surface is probably slightly different than the one analyzed by SEM-EDX and TEM-EDX almost completely covered by NaMgF_3_. For the sample analyzed by microscopy techniques, it is possible that the size of the brucite crystallites also decreases due to NaMgF_3_ precipitation. Indeed, generally, Mg(OH)_2_ is transformed to MgF_2_ and then to NaMgF_3_ in the longer term [15]. This point will be detailed below.

## 4. Discussion

### 4.1. Processes Occurring during MgZr Corrosion in Poral Solutions

**Figure 10:**
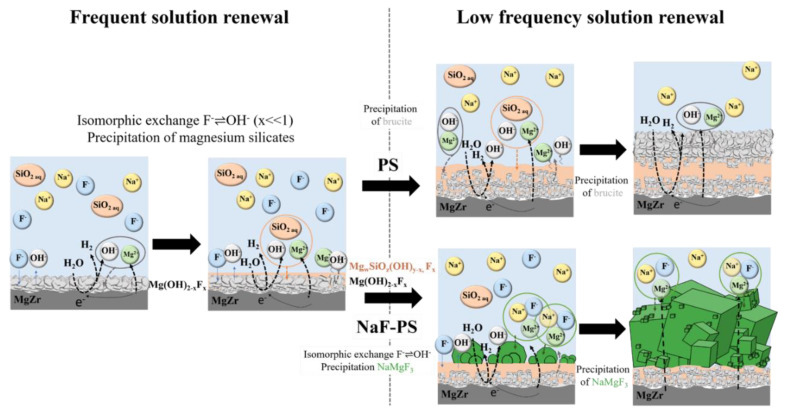
Diagram summarizing the possible processes which can occur during MgZr alloy corrosion in the experimental conditions of the study in PS and NaF-PS solutions extracted from geopolymers without and with NaF, respectively.

The characterization of the CP layers formed during the MgZr alloy corrosion in poral solutions extracted from geopolymers allowed the determination of various processes which depend on the elements available in solution such as dissolved silica SiO_2(aq)_ and F^−^ and thus, the renewal of the poral solution. These processes are summarized in Figure 10.

Whatever the poral solution, the processes occurring are close when the solution is frequently renewed, i.e., at the beginning of the experiment. Indeed, at the beginning of the MgZr corrosion Mg(OH)_2−*x*_F*_x_* is formed. The value of the subscript depends on the F concentration in solution. When F is present at a low concentration (PS), *x* << 1. When NaF is added during the geopolymer preparation and thus when F is at high concentration in poral solution, x is higher. Mg(OH)_2−*x*_F*_x_* is formed with the following processes. First, according to the pH > 10.5, a stable film of Mg(OH)_2_ should form according to (2):

Mg^2+^ + 2 H_2_O → Mg(OH)_2_ + 2H_2_ (2)

Thus, local dissolution of magnesium can also lead to the precipitation of Mg(OH)_2_ following (3):

Mg^2+^ + 2 OH^−^ → Mg(OH)_2_ (3)

Then, F incorporates in brucite lattice through an isomorphic exchange of OH^−^ with F^−^. Afterwards, magnesium silicates such as Mg*_w_*SiO*_z_*(OH)*_y_*_−*x*_F*_x_* (with *x* << 1 in PS and *x* >> 0 in NaF-PS) precipitate. The precipitation of these magnesium silicates is associated with a decrease of the size of brucite crystallites. Three hypotheses may explain this observation. First, to be formed, magnesium silicates consumed Mg from solution and from the brucite, leading to a decrease of crystallites size. Such interconnected processes have been already highlighted during glass alteration [27,28,29,30,31]. Second, the decrease of the Mg concentration in solution due to the magnesium silicates precipitation may generate the formation of crystallites having smaller size. Third, the possible protective properties of magnesium silicates regarding MgZr corrosion may limit the concentration of Mg in solution leading, here too, to the formation of crystallites having small sizes.

For longer corrosion duration, the solution renewal is less frequent in this experiment and thus, the dissolved silica supply being lower, the precipitation of magnesium silicates is limited. In that case, two processes can occur depending on the concentration of F in solution.

When the concentration of F is low, as in PS, brucite precipitates. The platelet morphology observed, typical of brucite is characteristic of precipitation and grain growth with the dissolution of high energy surfaces and development of low energy basal surface observed at pH above 12 [23].

When the concentration of F is high enough as in NaF-SP, NaMgF_3_ precipitates. Such precipitation can be explained by a high substitution of OH^−^ by F^−^ in Mg(OH)_2−*x*_F*_x_* leading to the formation of MgF_2_, not observed in this study, and then NaMgF_3_ as in [18]. Indeed, several studies [32] highlighted that the formation of crystalline NaMgF_3_ comes from the precipitation of nanospheres of MgF_2_ which react with NaF existing in solution at a higher concentration as described in (4) to form NaMgF_3_ [16,33]:

MgF_2_ +NaF → NaMgF_3_ (4)

Others studies [32,33,34] have shown that the cubic morphology of NaMgF_3_ is different from that of MgF_2_. Generally, MgF_2_ precipitate consists of spherical nanoparticles but this morphology can be modified as a function of temperature, the pH and the ration F/Mg [35,36].

### 4.2. Is There a Relation between the Passivating Properties of CP Layers and Their Morphology and Structure?

In this study, it is not possible to estimate the corroded MgZr volume and thus, to compare the impact of the poral solution used. Indeed, the thickness of the CP layers formed in PS or NaF-PS are close (1.42 vs. 1.60 µm) and their formation are not isovolumic regarding MgZr corrosion. The volume occupied by the phases depends mainly on their crystalline structures. However, the presence of various phases in CP layers may decrease the MgZr corrosion depending on their porous texture and stability in solution. Indeed, several studies performed in basic solutions have highlighted the passivating properties of CP layers formed in the presence of corrosion inhibitor such as silicates [10,19] and F^−^ [13,14,16,37] and their synergic effect when they are mixed together [18,19]. In these experiments, magnesium silicates and Mg(OH)_2−*x*_F*_x_* may passivate the MgZr surface.

Magnesium silicates and brucite are known to reduce the magnesium corrosion [3,7,8,9]. Nevertheless, such phases can present various and complex porous form. This is the case of brucite [38,39,40,41] modifying its passivating properties. In our experiment, the brucite formed in PS at the surface of MgZr presents an amorphous character and a denser texture than the brucite formed at the surface of the magnesium silicates having a platelet shape (Figure 3). Being denser, the brucite formed at the first time of the experiment may limit the solution transport through the CP layer and may be more protective regarding the corrosion of MgZr.

Fluorine in solution may be of interest since the numerous substitutions of OH^−^ by F^−^ forming Mg(OH)_2−*x*_F*_x_* lead to a decrease of the cell volume [42] and thus, to a possible densification of the substituted brucite [16]. Such densification may limit, here too, the solution transport through the CP layer and may decrease the MgZr corrosion. Moreover, the presence of fluorine in substituted brucite leads to a decrease of its dissolution and then improve the stability of the passivating effect [43]. As an example, MgF_2_ has a dissolution rate 10 times lower than brucite in basic media [44]. Rooses et al. have reported that the thickness calculated from the release of H_2_ during MgZr corrosion is two times lower in synthetic poral solution from NaF-GP than from GP. In that case, the presence of a passivating layer of Mg(OH)_2−*x*_F*_x_* with *x* >> 0 may explain such result. (References [45,46,47,48] are cited in the “Supplementary Materials”).

In addition, the formation of NaMgF_3_ from MgF_2_ may seal the porosity of the inner layer of Mg(OH)_2−*x*_F*_x_*, reducing the diffusion of species.

## 5. Conclusions

In this study, we investigated the evolution of the CP formed during the corrosion of MgZr in poral solutions extracted from geopolymers with and without NaF as corrosion inhibitor. Using various characterization techniques, we showed that the amount of dissolved species in solution such as silica and fluoride is the key parameter driving the nature of CP formed.

Even if the nature of CP is well identified, their passivating properties have to be investigated quantifying the MgZr corrosion rate and characterizing their various porous texture. In addition, their stability in solution and thus, the preservation of the protective properties has to be assessed in order to be able to predict the MgZr corrosion. This is of interest to predict the H_2_ release coming from the corrosion of MgZr fuel cladding embedded in geopolymer in geological repository. To reach this goal, some experiments are ongoing to determine the relation among the porous texture, the protective properties and the stability of synthetic corrosion products, formed in this study, in poral solution derived from geopolymers.

## Figures and Tables

**Table 1:** Impurities in MgZr (data supplied by Neyco).

Impurities	Al	As	Co	Cr	Cu	Fe	Mn	Ni	Sb	Zn	Cl
Quantity (ppm)	<10	<20	<10	22	2	9	11	1	<10	31	10

**Table 2:** References, pH and composition of the solutions used in this study.

References	pH	[Na] mmol·L^−1^	[Si] mmol·L^−1^	[F] mmol·L^−1^	[Cl] mmol·L^−1^
PS	12.37	418.9 ± 6.5	46.6 ± 15.8	19.0 ± 0.4	27.4 ± 0.5
NaF-PS	12.40	1116.5 ± 237.8	32.9 ± 9.1	1855.9 ± 37.1	20.0 ± 0.4

## Supplementary Materials

The following are available online at https://www.mdpi.com/1996-1944/13/21/4958/s1, Figure S1: Scheme of one pore in contact with MgZr substrate; S: Surface of Mg-Zr, V: Volume of the pore, Figure S2: Evolution of S/V calculated for half-spherical and half-cylindrical pores, Figure S3: Penetration depth P as a function of incident angle θ*_i_* for several compounds, Figure S4: GI-XRD cell made of half ping-pong ball sealed with CAF 4^®^, Figure S5: EDX cartographies of Na and C of the corroded MgZr substrates in (a) PS and (b) NaF-PS as a function of corrosion time, Figure S6: EDX average spectra of cartographies of the corroded MgZr substrates in (a) PS and (b) NaF-PS as a function of corrosion time, Figure S7: EDX spectra of the 2 different zones of the corroded MgZr substrate in NaF-PS at 360 days.

## References

[1] Caillat R.H.J., Salesse M. (1963). Raisons du choix de l’alliage Mg–Zr pour le gainage des elements combustibles. J. Nucl. Mater..

[2] Benavent V., Frizon F., Poulesquen A. (2016). Effect of composition and aging on the porous structure of metakaolin-based geopolymers. J. Appl. Crystallogr..

[3] Perrault G.G. (1974). The potential-pH diagram of the magnesium-water system. J. Electroanal. Chem. Interfacial Electrochem..

[4] Li S., Bacco A.C., Birbilis N., Cong H. (2016). Passivation and potential fluctuation of Mg alloy AZ31B in alkaline environments. Corros. Sci..

[5] Lambertin D., Frizon F., Bart F. (2012). Mg–Zr alloy behavior in basic solutions and immobilization in Portland cement and Na-geopolymer with sodium fluoride inhibitor. Surf. Coat. Technol..

[6] Rooses A., Steins P., Dannoux-Papin A., Lambertin D., Poulesquen A., Frizon F. (2013). Encapsulation of Mg–Zr alloy in metakaolin-based geopolymer. Appl. Clay Sci..

[7] Hsiao H.-Y., Tsung H.-C., Tsai W.-T. (2005). Anodization of AZ91D magnesium alloy in silicate-containing electrolytes. Surf. Coat. Technol..

[8] Wang J.-Y., Liu C.-M., Chen W.-K., Liu Y.-M., Ger M.-D. (2008). Microstructure and corrosion resistance of anodized Mg-9 mass% Li-1 mass% Zn alloy. Mater. Trans..

[9] Wu H.-L., Cheng Y.-L., Li L.-L., Chen Z.-H., Wang H.-M., Zhang Z. (2007). The anodization of ZK60 magnesium alloy in alkaline solution containing silicate and the corrosion properties of the anodized films. Appl. Surf. Sci..

[10] Hu J., Huang D., Song G.-L., Guo X. (2011). The synergistic inhibition effect of organic silicate and inorganic Zn salt on corrosion of Mg-10Gd-3Y magnesium alloy. Corros. Sci..

[11] Supplit R., Koch T., Schubert U. (2007). Evaluation of the anti-corrosive effect of acid pickling and sol–gel coating on magnesium AZ31 alloy. Corros. Sci..

[12] Lamaka S.V., Vaghefinazari B., Mei D., Petrauskas R.P., Höche D., Zheludkevich M.L. (2017). Comprehensive screening of Mg corrosion inhibitors. Corros. Sci..

[13] El-Taib Heakal F., Tantawy N.S., Shehata O.S. (2012). Impact of chloride and fluoride additions on surface reactivity and passivity of AM60 magnesium alloy in buffer solution. Corros. Sci..

[14] El-Taib Heakal F., Fekry A.M., Fatayerji M.Z. (2009). Influence of halides on the dissolution and passivation behavior of AZ91D magnesium alloy in aqueous solutions. Electrochim. Acta.

[15] Bradford P.M., Case B., Dearnaley G., Turner J.F., Woolsey I.S. (1976). Ion beam analysis of corrosion films on a high magnesium alloy (Magnox Al 80). Corros. Sci..

[16] Gulbrandsen E., Taftø J., Olsen A. (1993). The passive behaviour of Mg in alkaline fluoride solutions. Electrochemical and electron microscopical investigations. Corros. Sci..

[17] Gao H., Li Q., Chen F.N., Dai Y., Luo F., Li L.Q. (2011). Study of the corrosion inhibition effect of sodium silicate on AZ91D magnesium alloy. Corros. Sci..

[18] Barros C.F., Muzeau B., L’Hostis V., François R. (2020). Impact of fluoride concentration on general corrosion of Mg–Zr alloy in a Na-geopolymer and alkaline solutions. Corros. Sci..

[19] Rooses A., Lambertin D., Chartier D., Frizon F. (2013). Galvanic corrosion of Mg–Zr fuel cladding and steel immobilized in Portland cement and geopolymer at early ages. J. Nucl. Mater..

[20] Cyr M., Rivard P., Labrecque F., Daidié A. (2008). High-pressure device for fluid extraction from porous materials: Application to cement-based materials. J. Am. Ceram. Soc..

[21] Dahmen U., Kim M.G., Searcy A.W. (1987). Microstructural evolution during the decomposition of Mg(OH)_2_. Ultramicroscopy.

[22] Song G.-L., Unocic K.A. (2015). The anodic surface film and hydrogen evolution on Mg. Corros. Sci..

[23] Maltseva A., Shkirskiy V., Lefèvre G., Volovitch P. (2019). Effect of pH on Mg(OH)_2_ film evolution on corroding Mg by in situ kinetic Raman mapping (KRM). Corros. Sci..

[24] Akao M., Iwai S. (1977). The hydrogen bonding of hydromagnesite. Acta Crystallogr. Sect. B Struct. Crystallogr. Cryst. Chem..

[25] Mellini M.Z., Zanazzi P.F. (1987). Crystal structures of lizardite-1T and lizardite-2H1 from Coli, Italy. Am. Mineral..

[26] Wei M., Evans J.H., Bostrom T., Grondahl L. (2003). Synthesis and characterization of hydroxyapatite, fluoride-substituted hydroxyapatite and fluorapatite. J. Mater. Sci. Mater. Med..

[27] Ribet S., Gin S. (2004). Role of neoformed phases on the mechanisms controlling the resumption of SON68 glass alteration in alkaline media. J. Nucl. Mater..

[28] Michelin A., Burger E., Rebiscoul D., Neff D., Bruguier F., Drouet E., Dillmann P., Gin S. (2013). Silicate glass alteration enhanced by iron: Origin and long-term implications. Environ. Sci. Technol..

[29] Aréna H., Godon N., Rébiscoul D., Podor R., Garcès E., Cabie M., Mestre J.P. (2016). Impact of Zn, Mg, Ni and Co elements on glass alteration: Additive effects. J. Nucl. Mater..

[30] Aréna H., Godon N., Rébiscoul D., Frugier P., Podor R., Garcès E., Cabie M., Mestre J.P. (2017). Impact of iron and magnesium on glass alteration: Characterization of the secondary phases and determination of their solubility constants. Appl. Geochem..

[31] Rébiscoul D., Tormos V., Godon N., Mestre J.P., Cabie M., Amiard G., Foy E., Frugier P., Gin S. (2015). Reactive transport processes occurring during nuclear glass alteration in presence of magnetite. Appl. Geochem..

[32] Hsu W.P., Zhong Q., Matijević E. (1996). The Formation of uniform colloidal particles of magnesium fluoride and sodium magnesium fluoride. J. Colloid Interface Sci..

[33] Zuleta A.A., Correa E., Castaño J.G., Echeverría F., Baron-Wiecheć A., Skeldon P., Thompson G.E. (2017). Study of the formation of alkaline electroless Ni–P coating on magnesium and AZ31B magnesium alloy. Surf. Coat. Technol..

[34] Sevonkaev I., Goia D.V., Matijevic E. (2008). Formation and structure of cubic particles of sodium magnesium fluoride (neighborite). J. Colloid Interface Sci..

[35] Nandiyanto A.B., Iskandar F., Ogi T., Okuyama K. (2010). Nanometer to submicrometer magnesium fluoride particles with controllable morphology. Langmuir Acs J. Surf. Colloids.

[36] Xu Y.-W., Wang H.-Z. (2019). Preparation of sodium magnesium fluoride particles of different morphologies by EDTA-assisted hydrothermal method. J. Inorg. Mater..

[37] Ono S., Asami K., Masuko N. (2001). Mechanism of chemical conversion coating film growth on magnesium and magnesium alloys. Mater. Trans..

[38] Taheri M., Phillips R.C., Kish J.R., Botton G.A. (2012). Analysis of the surface film formed on Mg by exposure to water using a FIB cross-section and STEM–EDS. Corros. Sci..

[39] Brady M.P., Rother G., Anovitz L.M., Littrell K.C., Unocic K.A., Elsentriecy H.H., Song G.L., Thomson J.K., Gallego N.C., Davis B. (2015). Film breakdown and nano-porous Mg(OH)_2_ formation from corrosion of magnesium alloys in salt solutions. J. Electrochem. Soc..

[40] Das P.S., Dey A., Mandal A.K., Dey N., Mukhopadhyay A.K. (2013). Synthesis of Mg(OH)_2_ micro/nano flowers at room temperature. J. Adv. Ceram..

[41] Phillips V.A., Opperhauser H., Kolbe J.L. (1978). Relations among particle size, shape, and surface area of Mg(OH)_2_ and its calcination product. J. Am. Ceram. Soc..

[42] Ribbe R.H. (1979). Titanium, fluorine, and hydroxyl in the humite minerals. Am. Miner..

[43] Vermilyea D.A., Kirk C.F. (1969). Studies of inhibition of magnesium corrosion. J. Electrochem. Soc..

[44] Pokrovsky O.S., Schott J., Castillo A. (2005). Kinetics of brucite dissolution at 25 °C in the presence of organic and inorganic ligands and divalent metals. Geochim. Et Cosmochim. Acta.

[45] Vineyard H. (1982). Grazing-incidence diffraction and the distorted-wave approximation for the study of surfaces. Phys. Rev. B.

[46] Singh M., Manurung P., Low I.M. (2002). Depth profiling of near-surface information in a functionally graded alumina/aluminium titanate composite using grazing-incidence synchrotron radiation diffraction. Mater. Lett..

[47] Henke B.L., Gullikson E.M., Davis J.C. (1993). X-ray interactions: Photoabsorption, scattering, transmission, and reflection at E = 50–30, 000 eV, Z = 1–92. At. Data Nucl. Data Tables.

[48] X-Ray Attenuation Length. http://henke.lbl.gov/optical_constants/atten2.html.

