# Supplementary material for: Evolution of Corrosion Products Formed during the Corrosion of MgZr Alloy in Poral Solutions Extracted from Na-Geopolymers Used as Conditioning Matrix for Nuclear Waste
Source: Materials (Basel). 2020 Nov 4;13(21):4958. doi: 10.3390/ma13214958 (PMC7663393; doi:10.3390/ma13214958)
Supplement: Supplementary file 1 [file materials-13-04958-s001.pdf]

Supplementary Materials: Evolution of Corrosion Products Formed during the Corrosion of MgZr Alloy in Poral Solutions Extracted from Na-Geopolymers Used as Conditioning Matrix for Nuclear Waste

Rémi Boubon [1], Xavier Deschanels [1], Martiane Cabié [2] and Diane Rébiscoul [1,*]

[1] ICSM, CEA, CNRS, ENSCM, Univ Montpellier, Marcoule, 30207 Bagnols-sur-Cèze, France; remi.boubon@cea.fr (R.B.); xavier.deschanels@cea.fr (X.D.)
[2] CP2M, Aix-Marseille Université, 13397 Marseille, France; martiane.cabie@univ-amu.fr
* Correspondence: diane.rebiscoul@cea.fr

S/V calculation

Geopolymers are nanoporous materials having pores sizes from 2 to 15 nm. The S/V ratio is the ratio between the surface of the metal in contact with a volume of solution as schematize in **Error! Reference source not found.**.

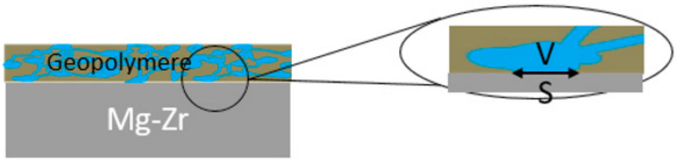

Figure S1. Scheme of one pore in contact with MgZr substrate; S: Surface of MgZr, V: Volume of the pore.

By doing the assumption of having half-spherical or half-cylindrical pores and taking different size of pore as characterized with a similar composition of GP [2]. The evolution of this ratio is presenting in Figure S2. It goes from 10^{11} to 10^{10} m^{-1} but we can estimate it around 10^{10} m^{-1}.

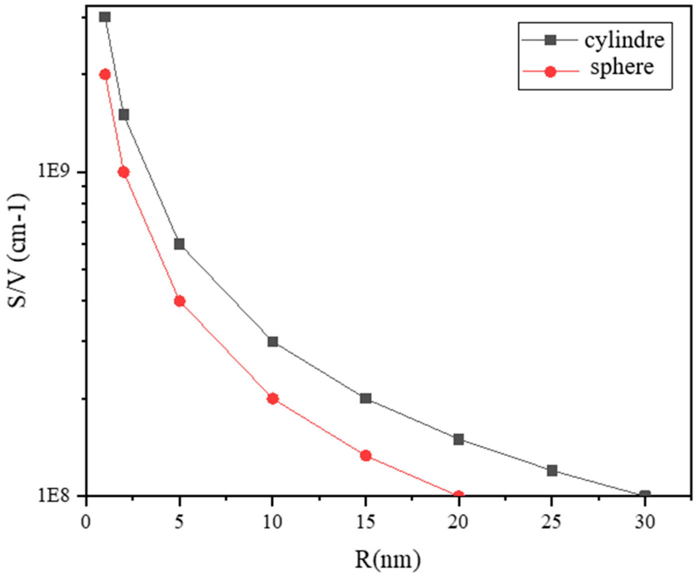

Figure S2. Evolution of S/V calculated for half-spherical and half-cylindrical pores.

Penetration depth calculation

The penetration depth of the X-ray beam $P(\theta i)$(nm) is written as Equation (1) [45]:

$$P(\theta i) = \frac{\lambda\left[\left[(\theta i^2 - \theta_c{}^2)^2 + 4\beta^2\right]^{1/2} + \left((\theta i^2 - \theta_c^2)\right]^{1/2}}{4(2^{1/2})\beta\pi} \quad (1)$$

with λ the wavelength, d the interreticular distance, θ_c the critical angle for a total reflexion of the X-ray beam [46] Equation (2) and β the absorption coefficient of the material [47].

$$\theta_c \approx (2\delta)^{1/2} = 1.6 \times 10^{-3}\ \rho\lambda \quad (2)$$

with δ =1−n and n the refractive index of the material.

The Figure S3 presents the variation of $P(\theta i)$ considering the formation of various magnesium compounds ($Mg(OH)_2$, MgO, MgF_2...). Data calculated from the B.L. Henkel calculator [48] from data of [47].

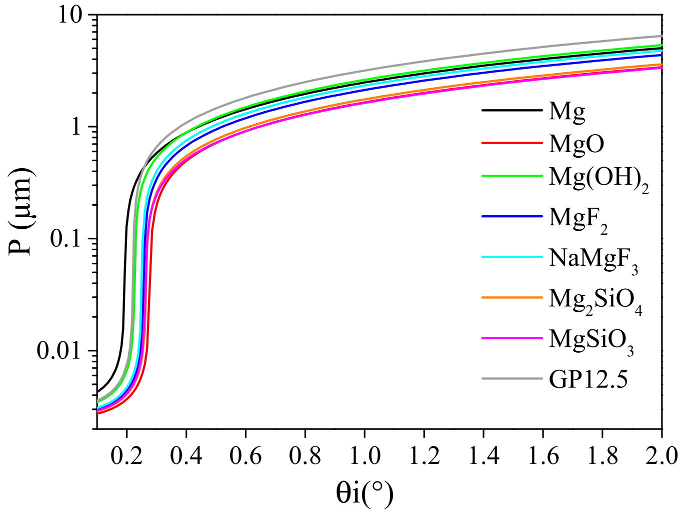

Figure S3. Penetration depth P as a function of incident angle θ_i for several compounds.

GI-XRD Measurement cells

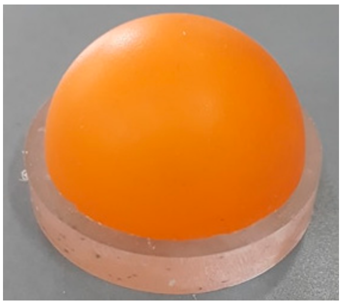

Figure S4. GI-XRD cell made of half ping-pong ball sealed with CAF 4®.

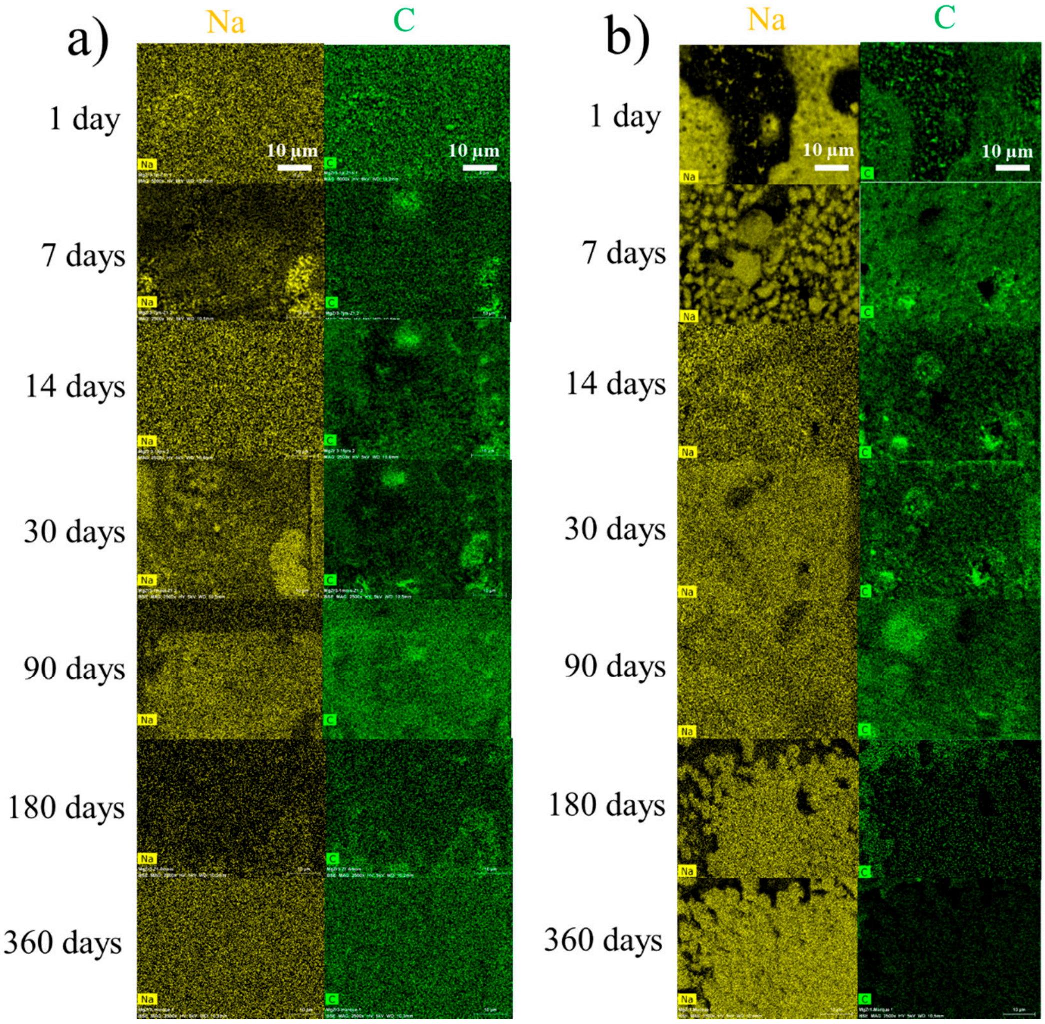

Figure S5. EDX cartographies of Na and C of the corroded MgZr substrates in (**a**) PS and (**b**) NaF-PS as a function of corrosion time.

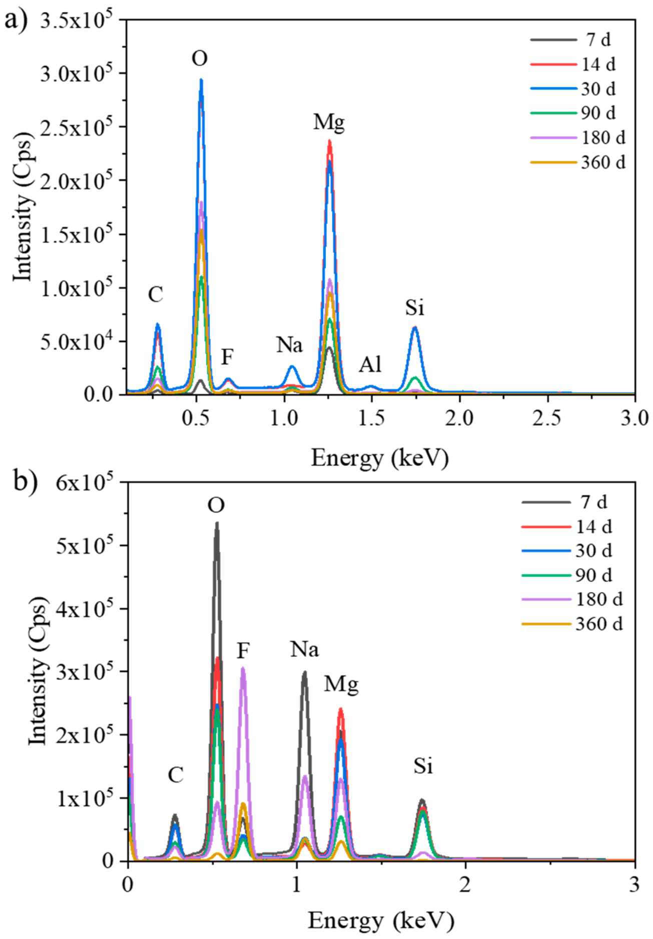

Figure S6. EDX average spectra of cartographies of the corroded MgZr substrates in (**a**) PS and (**b**) NaF-PS as a function of corrosion time.

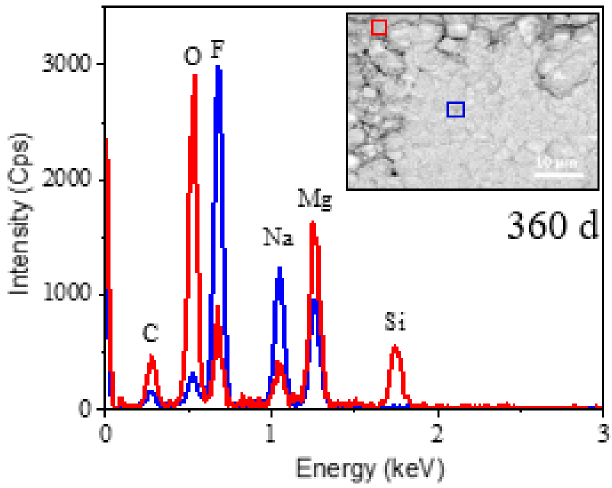

Figure S7. EDX spectra of the two different zones of the corroded MgZr substrate in NaF-PS at 360 days.

References of SI

References are provided in the full paper.

Publisher's Note: MDPI stays neutral with regard to jurisdictional claims in published maps and institutional affiliations.

© 2020 by the authors. Submitted for possible open access publication under the terms and conditions of the Creative Commons Attribution (CC BY) license (http://creativecommons.org/licenses/by/4.0/).
